# Changes of Composition and Nutrition Value of Some Gluten Free (Pseudo)cereals by Addition of Carotenogenic Yeast Cell Homogenate

**DOI:** 10.3390/molecules31091460

**Published:** 2026-04-28

**Authors:** Agáta Bendová, Paula Večeríková, Pavlína Sniegoňová, Jan Obračaj, Jiří Holub, Ivana Márová

**Affiliations:** Faculty of Chemistry, Brno University of Technology, Purkyňova 464/118, 612 00 Brno, Czech Republic; xcbendova@fch.vut.cz (A.B.);

**Keywords:** (pseudo)cereals, carotenogenic yeast, *Rhodotorula toruloides*, enrichment

## Abstract

Gluten-free cereals and pseudocereals such as oats, quinoa, and buckwheat are widely used as bases for functional foods due to their protein quality, minerals, fiber, and polyphenols. However, they contain relatively low levels of some vitamins. This study evaluated the enrichment of cereal porridges with a cell-free homogenate of the carotenogenic yeast *Rhodotorula toruloides* (RT) as a natural strategy to enhance their nutritional value. Model porridges prepared from gluten-free oats, quinoa, buckwheat, and their blends were supplemented with 0%, 5%, or 10% *R. toruloides* homogenate (RTh). Samples were analyzed for antioxidant capacity (ABTS), lipid-soluble vitamins and provitamins (HPLC), fatty acid composition (GC-FID), approximate prebiotic potential, and cytotoxicity using the MTT assay on Caco-2 cells. The addition of RTh significantly increased antioxidant properties, with the highest value observed in buckwheat porridge with 5% RTh (1.9 mg TE/g DW). Lipid-soluble metabolites were detected only in enriched samples, reaching up to 420 µg/g DW ergosterol and 300 µg/g DW total carotenoids, mainly torularhodin. Quinoa porridges showed the highest PUFA content, whereas RTh was rich in oleic acid. Enrichment increased MUFA levels and improved the fatty acid profile. Approximate prebiotic potential was strongest in oat–quinoa blends. Cytotoxicity remained low, with most IC_50_ values above 2000 µg/mL. Supplementation with 5% *R. toruloides* homogenate effectively improves antioxidant and lipid composition of gluten-free porridges while maintaining low cytotoxicity.

## 1. Introduction

Cereal grains are among the most important staple foods worldwide, providing carbohydrates, dietary fiber, plant proteins, antioxidants, and micronutrients to a large part of the global population [1,2]. In addition to true cereals, pseudocereals such as quinoa and buckwheat are increasingly valued as nutrient-dense, gluten-free alternatives rich in bioactive compounds. Due to their adaptability to unfavorable conditions, pseudocereals are considered promising crops for the future [3,4].

Beyond their nutritional role, cereals are widely applied in functional food development, as non-digestible carbohydrate sources can exert prebiotic effects, support probiotic growth, and supply dietary fiber with physiological benefits [5,6]. Global cereal availability, nutrient density, long shelf life, and high consumption frequency make cereals ideal carriers for fortification, and enriched products have been shown to improve micronutrient intake and support public health. Moreover, regular consumption of whole grains is consistently associated with reduced risks of chronic diseases, including cardiovascular disease, type 2 diabetes, and certain cancers [7,8].

Celiac disease is a chronic autoimmune disorder triggered by gluten proteins from wheat, rye, and barley, affecting approximately 1% of the global population. The only effective treatment is a strict lifelong gluten-free diet [9,10]. Gluten-free cereals and pseudocereals, including oats, quinoa, and buckwheat, therefore represent essential food resources for affected individuals and for consumers seeking healthier alternatives [4,11].

Oats (*Avena sativa* L.), quinoa (*Chenopodium quinoa*) and buckwheat (*Fagopyrum* spp.) are nutritionally balanced cereals and pseudocereals containing about 10–17% of protein, 5–13% of lipids, and 60–75% of carbohydrates. Oats, quinoa and buckwheat are also sources of polyphenols, flavonoids, and minerals [12,13,14]. Oats are particularly valued for soluble β-glucans (1.8–7% DW), which contribute to lowering LDL cholesterol and improving glycemic control, with EFSA and FDA recognizing that ≥3 g/day supports normal blood cholesterol levels. Pure oats are also generally tolerated by most individuals with celiac disease if contamination with gluten cereals is avoided [15,16,17].

Cereal fibers contain various non-starch polysaccharides that are fermented in the colon to produce short-chain fatty acids (SCFAs) such as butyrate (fuel for colon cells), acetate and propionate. SCFAs improve gut barrier function, immunity, and metabolic health [18]. Cereal fibers have high prebiotic activity due to the high content of arabinoxylans, β glucans, resistant starch, fructans (inulin-type fibers), cellulose and lignin (insoluble fibers). Cereal fibers—especially arabinoxylans, β glucans, and resistant starch—function as powerful prebiotics that support beneficial gut microbiota and gastrointestinal and metabolic health. Some of the above-mentioned types of non-digestible carbohydrates that show strong prebiotic activity, meaning they can stimulate beneficial bacteria in the gut, contain quinoa, buckwheat and oat [18,19,20]. A strategy to maximize the prebiotic effect is to cook or cool the grains, combine them with other (pseudo)cereals or legumes, and consume them in their unprocessed whole grain form [21,22].

Nevertheless, as mentioned above, pseudocereals (quinoa, buckwheat) and some cereals (oats) are nutritionally valuable, especially for protein quality, minerals, fiber, and polyphenols, but they are not rich sources of many vitamins. Compared to true cereals or vegetables, their vitamin content is generally low. Expect for a few cases, there are low levels of several B vitamins, typically vitamin B_2_, B_3_ and B_6_. Vitamin B_12_ is absent (present only in animal-derived foods), while folate is moderate in quinoa, lower in others, but still not high enough to cover daily needs. Like all plant foods, pseudocereals contain no vitamin D, and very low vitamin A/carotenoids. Like all grains, (pseudo)cereals contain no vitamin C [23,24,25].

If we want to take advantage of the nutritional value and prebiotic effect of cereals, one strategy is to fortify them with the vitamins and other compounds they lack. As a simple one-step fortification by a whole complex of vitamins, provitamins and other valuable nutrients, the use of homogenate of some yeast cell species could be a promising alternative. After cultivation, yeast cell biomass could be homogenized and the cell-free homogenate can be directly added to cereal or pseudocereal products.

The most frequently used yeasts in the food and feed industry strains of *Saccharomyces* sp. are applied. Although *Saccharomyces cerevisiae* has been reported to cause allergic reactions in rare cases, particularly in highly atopic individuals [26], yeast-derived products are generally well tolerated in food applications. Nevertheless, in recent years, non-conventional yeasts have progressively transitioned from biotechnological production platforms toward applications in human nutrition. A prominent example is *Yarrowia lipolytica*, whose dried yeast biomass has been authorized in the European Union as a novel food following comprehensive safety evaluation by the EFSA. The initial authorization and subsequent extensions of use, as reflected in the Union list of novel foods, established a regulatory precedent for the utilization of whole yeast biomass as a food ingredient [27,28].

Beyond *Y. lipolytica*, carotenogenic yeasts have gained increasing attention as a promising source of nutritionally valuable compounds. Carotenogenic yeasts can accumulate nutritionally valuable metabolites such as lipids, carotenoids, ergosterol, and glucans [29]. Typical composition of red yeast cells ranges within: proteins (25–30% of CDW), intracellular lipids (20–66% of CDW), carotenoids (0.5–4.6 mg/g), ergosterol (1.2–4.6 mg/g), coenzyme Q_10_ (2.5.–6.8 mg/g), β–glucans (11–18% of CDW), with a biomass content of 15–20 g/L [29].

Among carotenogenic yeasts, the oleaginous yeast *Rhodotorula toruloides* has emerged as a particularly versatile microbial cell factory for functional food applications. In contrast to conventional yeasts such as *Saccharomyces cerevisiae*, which are widely used in food systems but exhibit limited production of lipophilic bioactive compounds, *R. toruloides* is characterized by the simultaneous synthesis of multiple nutritionally relevant metabolites. Specifically, *R. toruloides* naturally produces high amounts of carotenoids, including β-carotene, torulene, and torularhodin, which contribute to antioxidant activity and serve as provitamin A sources [30,31]. In addition, its biomass contains ergosterol, a precursor of vitamin D_2_ that can be converted via UV irradiation [32]. Furthermore, *R. toruloides* is among the most oleaginous yeasts, capable of accumulating 50–70% of its dry cell weight as lipids, predominantly unsaturated fatty acids such as oleic, linoleic, and α-linolenic acids [33,34]. While cereals and pseudocereals already contain moderate amounts of lipids (typically 5–13% of dry weight), these are quantitatively limited and their fatty acid profile is matrix-dependent [22,35]. Therefore, fortification with oleaginous yeast biomass represents an effective strategy to enhance both the total lipid content and the diversity of polyunsaturated fatty acids in cereal-based products [33,36]. In addition to its lipid and carotenoid profile, the cell wall of *R. toruloides* is rich in β-(1→3)(1→6)-glucans, which have been associated with immunomodulatory and functional health effects [29]. The combination of these properties makes *R. toruloides* a multifunctional fortification agent that provides a broader spectrum of bioactive compounds compared to commonly used yeast species. These characteristics distinguish *R. toruloides* from other non-conventional yeasts such as *Yarrowia lipolytica*, which, although efficient in lipid accumulation, lacks significant carotenoid production.

Biochemical composition of *R. toruloides* cell-free homogenate (water extract) and the growing body of biotechnological research suggest a potential for future nutritional applications, provided that appropriate safety assessments are conducted, similarly to the regulatory pathway previously established for *Y. lipolytica*. Furthermore, the potential use of *R. toruloides* biomass as a functional ingredient in cereal-based foods is supported by animal feeding trials reporting no adverse effects [37,38].

The aim of this study was to evaluate the effect of *Rhodotorula toruloides* cell-free homogenate (0–10%) on the nutritional composition, antioxidant capacity, lipid-soluble bioactive compounds, prebiotic potential, and cytotoxicity of gluten-free cereal-based porridges formulated from oat, buckwheat, and quinoa.

## 2. Results

The results presented below are based on the analysis of gluten-free cereal porridges enriched with 0%, 5%, and 10% of *Rhodotorula toruloides* cell-free homogenate. The samples were evaluated in terms of nutritional composition, including β-glucan content (Megazyme assay), lipid-soluble bioactive compounds (HPLC/PDA), and fatty acid profile (GC-FID), as well as antioxidant activity (ABTS assay), prebiotic potential using selected probiotic strains, and cytotoxicity on Caco-2 cells (MTT assay). All analyses were performed in triplicate, and the results are expressed as mean ± standard deviation.

### 2.1. Composition of Extracts

A number of metabolic parameters were determined in extracts of the selected (pseudo)cereals themselves and some cereal blends, and in extracts of mixtures of cereals with the red yeast cell-free homogenate in order to characterize their composition and the content of active compounds.

#### 2.1.1. β-Glucans

The β-glucan content of the samples is presented in Table 1. Among the pure cereal and pseudocereal samples, the highest β-glucan concentration was observed in oat (7.78 ± 0.41 g/100 g DW), which is consistent with the known high content of mixed-linkage β-(1→3)(1→4)-glucans in oat cell walls. In contrast, buckwheat and quinoa contained only trace amounts of β-glucans (0.17 ± 0.01 and 0.27 ± 0.04 g/100 g DW, respectively). Among the cereal and pseudocereal mixtures, the β-glucan content varied substantially depending on the presence of oat in the formulation. Mixtures containing oat exhibited considerably higher β-glucan levels compared to combinations of pseudocereals alone. For example, the oat–buckwheat and oat–quinoa mixtures contained approximately 3.98 ± 0.20 and 4.02 ± 0.20 g/100 g DW, respectively, whereas the buckwheat–quinoa mixture contained only 0.22 ± 0.01 g/100 g DW. The β-glucan content of the *R. toruloides* homogenate reached 16.45 ± 0.10 g/100 g DW, reflecting the typical abundance of β-(1→3)(1→6)-glucans in yeast cell walls. Due to structural differences in β-glucans present in cereals (β-(1→3)(1→4)) and yeast (β-(1→3)(1→6)), two different Megazyme kits were used for their determination. As a result, the β-glucan content in cereal–yeast mixtures could not be measured directly. Therefore, the values for the mixtures were calculated based on the measured β-glucan content of individual components, assuming proportional contributions. For these calculated mixtures, a standard deviation of approximately 5% was applied (Table 1). It should be noted that the estimated β-glucan values for cereal–yeast mixtures may influence the accuracy of quantitative comparisons.

Enrichment of cereal matrices with *R. toruloides* homogenate (RTh) resulted in a gradual increase in the calculated β-glucan content across all formulations. For example, oat-based samples increased from 7.78 g/100 g DW to 8.60 and 9.42 g/100 g DW after the addition of 5% and 10% RTh, respectively. A similar trend was observed in other cereal combinations, including oat–buckwheat and oat–quinoa mixtures, where β-glucan levels increased proportionally with the amount of yeast biomass added.

Overall, the results demonstrate that the incorporation of *R. toruloides* biomass effectively increases the β-glucan content of cereal-based formulations, particularly in matrices naturally rich in β-glucans such as oat, thereby potentially enhancing their functional and nutritional value.

#### 2.1.2. Lipid-Soluble Vitamins and Provitamins

**Figure 1:**
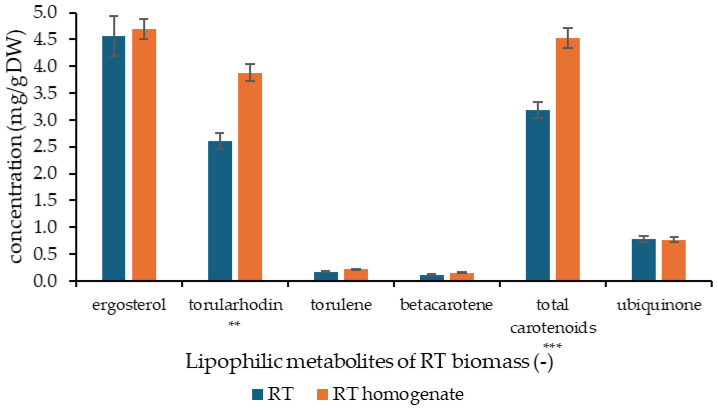
Content of lipid-soluble metabolites in *R. toruloides* biomass; the results were expressed as mg of lipophilic metabolites per g of dry weight of biomass; RT–non-treated biomass, RTh–cell homogenate; values are expressed as mean ± SD (n = 3). Asterisks indicate significant differences between RT and RTh (** *p* < 0.01, *** *p* < 0.001).

The analysis of lipid-soluble metabolites in *R. toruloides* biomass extracts revealed high levels of ergosterol and carotenoids (Figure 1). In non-treated biomass (RT), ergosterol reached 4.57 ± 0.38 mg/g DW, while in the cell homogenate (RTh) the content slightly increased to 4.70 ± 0.18 mg/g DW. A more notable difference was observed in torularhodin, which increased from 2.60 ± 0.15 mg/g DW in RT to 3.88 ± 0.16 mg/g DW in RTh. Similarly, torulene and β-carotene were detected at lower concentrations, but both showed higher values in the inactivated samples (0.17 ± 0.01 vs. 0.22 ± 0.00 mg/g DW for torulene; 0.12 ± 0.01 vs. 0.15 ± 0.01 mg/g DW for β-carotene). The concentration of total carotenoids increased from 3.18 ± 0.15 mg/g DW in RT to 4.53 ± 0.18 mg/g DW in RTh. Ubiquinone was present at comparable levels in both sample types, with 0.78 ± 0.06 mg/g DW in RT and 0.76 ± 0.04 mg/g DW in RTh.

Results revealed significant differences between RT and RT homogenate in torularhodin (*p* < 0.01) and total carotenoid content (*p* < 0.001), with higher values observed in RT homogenate. In contrast, no significant differences were detected for ergosterol and ubiquinone (*p* > 0.05). Minor carotenoids, including torulene and β-carotene, showed only slight, non-significant increases after homogenization.

These results suggest that cell disruption enhances the extractability or availability of carotenoids, while sterol and ubiquinone levels remain unaffected.

Lipid-soluble metabolites, including ergosterol and carotenoids (torularhodin, torulene, and lycopene), were detected exclusively in samples enriched with RTh (Table 2), whereas the non-supplemented control porridge showed no detectable levels. These findings highlight the importance of fortifying (pseudo)cereals products with yeast biomass as a strategy to enhance their nutritional value. Ergosterol was the predominant sterol in all enriched samples, with concentrations ranging approximately from 200 to 435 µg/g DW, and showed a clear increase with higher RTh addition (type 3 vs. type 2). Total carotenoid content followed a similar trend, increasing with biomass dosage and reaching the highest levels in type 3 samples, particularly in mixed cereal formulations (oat–buckwheat, oat–quinoa, buckwheat–quinoa). Among individual carotenoids, torularhodin represented the major fraction in most enriched samples, whereas torulene concentrations were comparatively lower and remained relatively stable across different matrices and enrichment levels. Lycopene was consistently detected in all enriched samples at moderate levels (approximately 47–142 µg/g DW) and exhibited an increasing trend with higher biomass incorporation, especially in quinoa- and oat-based blends (Table 2).

#### 2.1.3. Lipids and Fatty Acids

**Figure 2:**
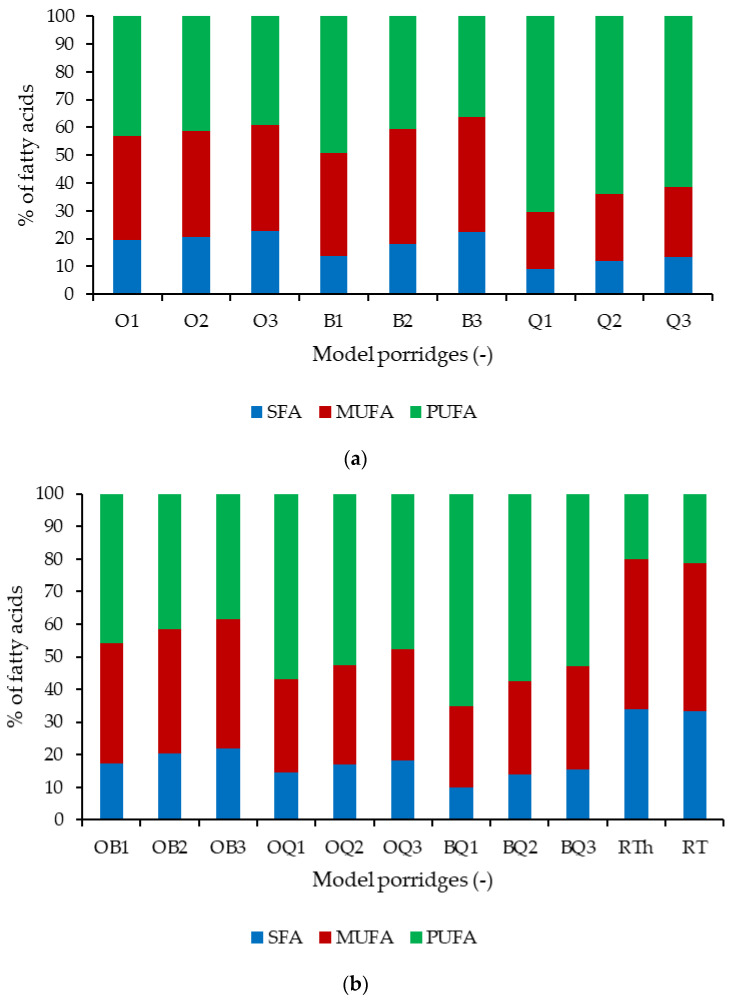
Percentage composition of fatty acids in model porridges; (**a**) single-component cereal bases, (**b**) two-component cereal bases; O—oat, B—buckwheat, Q—quinoa, OB—oat–buckwheat blend, OQ—oat–quinoa blend, BQ—buckwheat–quinoa blend, RTh—R. toruloides cell homogenate, RT—R. toruloides, type 1 = no RT fortification, type 2 = 5% RT fortification, type 3 = 10% RT fortification.

The fatty acid profiles of the model porridges varied according to the cereal base (Figure 2). Oat porridges (O1–O3) showed a balanced distribution of MUFA and PUFA, with moderate SFA levels. Buckwheat porridges (B1–B3) were characterized by the highest proportion of MUFA (up to 60%), while quinoa porridges (Q1–Q3) contained the highest share of PUFA (>60%) (Figure 2a). Mixed porridges (OB, OQ, BQ) reflected intermediate profiles, depending on the dominating cereal component (Figure 2b). Enrichment with RTh markedly altered the fatty acid composition by increasing SFA and MUFA while reducing PUFA, resulting in a profile that is more stable but less rich in essential fatty acids. Comparable proportions of saturated, monounsaturated, and polyunsaturated fatty acids were observed for RT and RTh, suggesting that cell homogenization and heat treatment had no major impact on the fatty acid composition (Figure 2b).

The fatty acid composition of the model porridges differed significantly depending on the cereal base (Table 3). Oat-based samples (O1–O3) contained the highest concentrations of oleic and linoleic acids, accompanied by moderate levels of palmitic acid. Buckwheat porridges (B1–B3) showed generally lower concentrations of all analyzed fatty acids, particularly linoleic acid, with stearic and α-linolenic acids present only at low levels. Quinoa-based porridges (Q1–Q3) were characterized by high linoleic acid contents (up to approximately 580 µg/mL) and a consistent presence of α-linolenic acid (approximately 35–40 µg/mL). Mixed cereal formulations reflected contributions from both components. Oat–buckwheat porridges (OB1–OB3) exhibited intermediate fatty acid levels with increased oleic acid compared to pure buckwheat samples. Oat–quinoa porridges (OQ1–OQ3) retained high linoleic acid concentrations and detectable α-linolenic acid, while buckwheat–quinoa porridges (BQ1–BQ3) showed higher linoleic acid levels than buckwheat alone but lower than quinoa-based samples. Fortification of cereal-based porridges with RTh led to an overall increase in the absolute concentrations of most analyzed fatty acids across all cereal matrices, with a more pronounced effect at 10% fortification compared to 5%. In particular, palmitic and stearic acids increased following RTh addition, and stearic acid became consistently detectable in enriched samples. Oleic acid showed the most pronounced increase upon RTh fortification in all formulations, whereas changes in linoleic acid depended on the cereal matrix. The content of α-linolenic acid generally increased in RTh-enriched samples, while the characteristic fatty acid patterns of individual cereal bases remained preserved. The fatty acid profile of RT and RTh differed from cereal-based porridges and was dominated by palmitic, stearic, and oleic acids, with linoleic acid present at intermediate levels and α-linolenic acid also detected. Lignoceric acid was detected exclusively in RT and RTh, while it was not detected in any of the cereal-based porridges. Comparable qualitative fatty acid patterns were observed for RT and RTh, indicating that cell homogenization and heat inactivation did not substantially alter the overall fatty acid composition (Table 3).

### 2.2. Characteristics of Cereals and Yeast Extracts

#### 2.2.1. Antioxidant Properties

**Figure 3:**
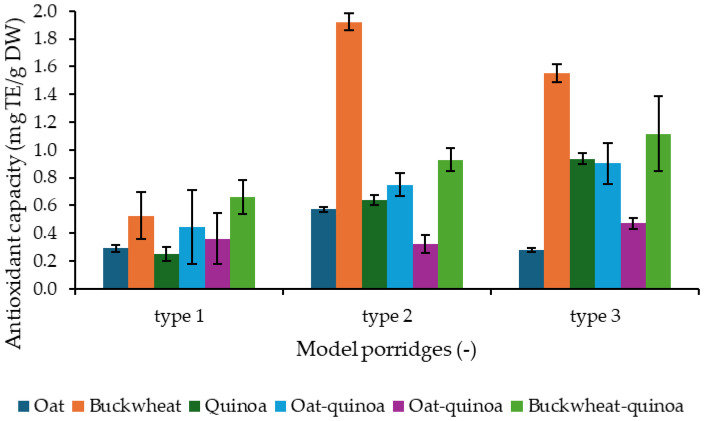
Determination of antioxidant capacity in model porridges; the results are expressed as mg of Trolox equivalents per g of dry weight of model porridges; type 1 = no RTh fortification, type 2 = 5% RTh fortification, type 3 = 10% RTh fortification.

The results of the antioxidant capacity analysis are presented in Figure 3. Across all model porridges, significant differences in antioxidant capacity were observed depending on the cereal base. The lowest values were consistently found in gluten-free oat porridge, while buckwheat showed the highest antioxidant capacity in all types of model porridges. Quinoa and the oat–quinoa and oat–buckwheat blends displayed intermediate activities, with the buckwheat–quinoa blend consistently outperforming oat and oat–buckwheat porridges. The strongest effect was observed in type 2 porridges, where buckwheat porridge reached nearly 1.9 mg TE/g DW.

The antioxidant capacity of RT differed markedly between the tested forms. Lyophilized cells showed substantially higher activity (5.47 ± 0.07 mg TE/g DW) compared to the cell homogenate (3.67 ± 0.17 mg TE/g DW).

Comparisons among the three types of model porridges showed that enrichment with RTh increased antioxidant capacity. Type 1 (0% RTh) generally had the lowest values. Type 2 (5% RTh) exhibited the highest antioxidant activities across most samples, with particularly strong effects in buckwheat and buckwheat–quinoa porridges. In type 3 (10% RTh), antioxidant activity remained elevated compared to type 1 but was in some cases lower than in type 2, indicating a non-linear dose–response (Figure 3).

#### 2.2.2. Approximate Prebiotic Potential

The approximate prebiotic potential of the model porridges was evaluated using the prebiotic index (PI), expressed as relative bacterial growth compared to MRS medium (Table 4). The non-fortified control samples (type 1) generally exhibited low to moderate approximate prebiotic potential, with several cereal matrices already supporting the growth of both *Lactobacillus plantarum* and *Bifidobacterium bifidum*.

Fortification with RTh markedly influenced the prebiotic response in a dose- and matrix-dependent manner. In samples fortified with 5% RTh (type 2), a pronounced increase in prebiotic index was observed for *L. plantarum*, with several formulations (oat + 5% RTh, buckwheat + 5% RTh, oat–buckwheat + 5% RTh, oat–quinoa + 5% RTh reaching high or very high prebiotic index categories (++ to +++). A similar but slightly less pronounced trend was observed for *B. bifidum*, where most type 2 samples exhibited moderate to high approximate prebiotic potential. In contrast, fortification at 10% RTh (type 3) resulted in more variable responses. While some matrices maintained high prebiotic potential, particularly toward *L. plantarum* (e.g., oat + 10% RTh, oat–buckwheat + 10% RTh, oat-quinoa + 10% RTh, a reduction in or absence of stimulation was observed for *B. bifidum* in several type 3 samples. The RTh alone showed limited approximate prebiotic potential, indicating that the observed effects in enriched porridges resulted from interactions between the cell homogenate and the cereal matrix rather than from RTh itself (Table 4).

The observed stimulation of bacterial growth indicates an approximate prebiotic potential; however, this conclusion is limited to the tested strains and does not represent the full complexity of the gut microbiota.

### 2.3. Cytotoxicity Measurement

**Figure 4:**
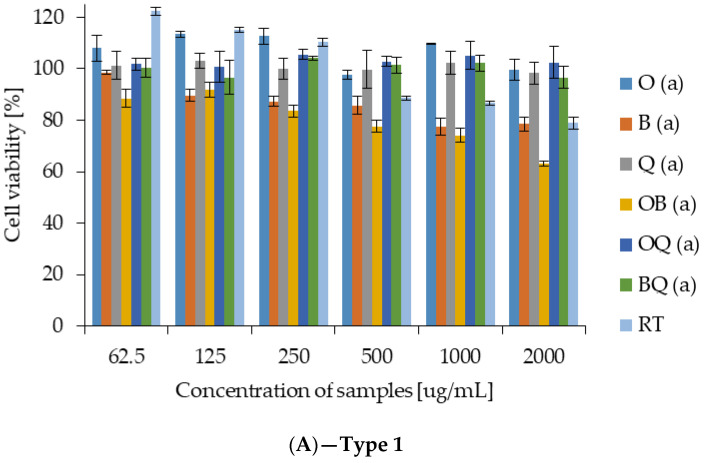

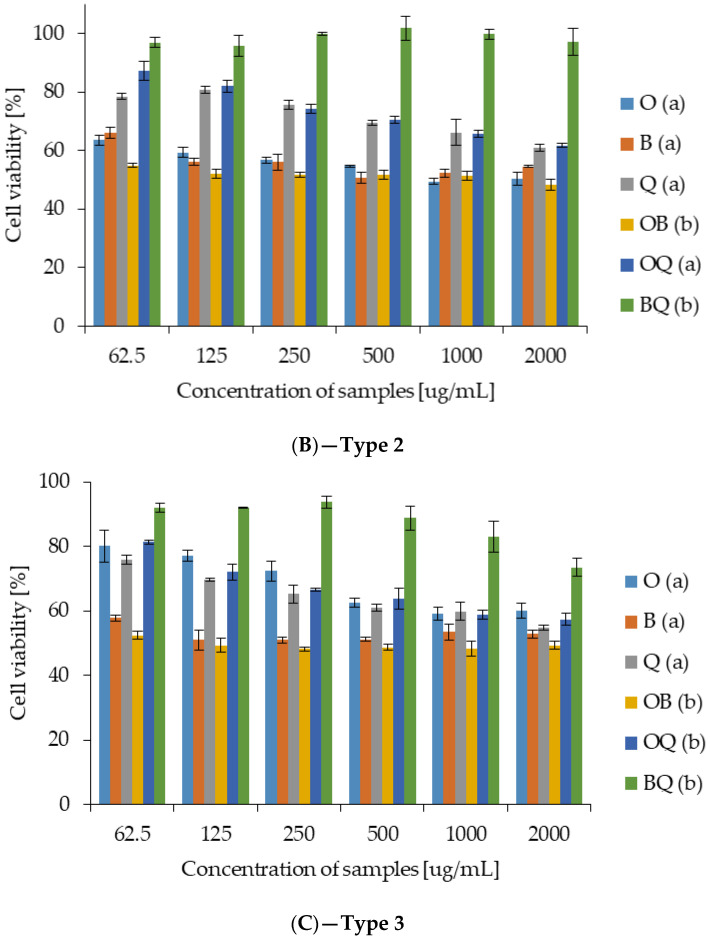
Determination of cell viability by MTT assay on Caco-2 cells (passages 50–60); (**A**) cell viability of type 1 porridges and RT cell homogenate, (**B**) cell viability of type 2 porridges, (**C**) cell viability of type 3 porridges; O—oat, B—buckwheat, Q—quinoa, OB—oat–buckwheat blend, OQ—oat–quinoa blend, BQ—buckwheat–quinoa blend, RT—*R. toruloides* cell homogenate, type 1 = no RTh fortification, type 2 = 5% RTh fortification, type 3 = 10% RTh fortification; different letters indicate statistically significant differences between samples (*p* < 0.05).

The MTT assay revealed distinct patterns of cell viability depending on the type of extract applied (Figure 4). Cytotoxicity was interpreted according to ISO 10993-5, where cell viability above 80% indicates no cytotoxicity, 60–80% mild cytotoxicity, 40–60% moderate cytotoxicity, and values below 40% severe cytotoxicity.

Across all tested concentrations, the RTh extract maintained relatively high cell viability, indicating no cytotoxic effect according to ISO 10993-5. Cell viability remained predominantly above 80%, even at the highest tested concentration of 2000 µg/mL, demonstrating good biocompatibility with the tested cells.

In type 1 samples (without RTh), most extracts showed no cytotoxicity, maintaining cell viability above 80% even at the highest tested concentration (2000 µg/mL). Only buckwheat and oat–buckwheat extracts caused a slight decrease in viability to 70–80%, which corresponds to mild cytotoxicity (Figure 4A). In type 2 samples (containing 5% RTh), a slight reduction in cell viability was observed. Oat, buckwheat and oat–buckwheat extracts reduced viability into the 60–80% range, indicating mild cytotoxicity, particularly at higher concentrations. In contrast, the buckwheat–quinoa combinations, maintained viability above 90%, indicating no cytotoxic effect (Figure 4B). In type 3 samples (10% RTh), the cytotoxic effect became more pronounced. Buckwheat and oat–buckwheat extracts reduced viability to approximately 50–60%, corresponding to moderate cytotoxicity according to ISO 10993-5. Oat and quinoa extracts showed mild cytotoxicity (60–70% viability), while buckwheat–quinoa combination again exhibited the highest viability (80–90%), indicating no or only negligible cytotoxicity. (Figure 4C).

The effect of *Rhodotorula toruloides* supplementation on Caco-2 cell viability was found to be strongly matrix-dependent. No statistically significant differences were observed in individual cereal samples (oat, buckwheat, quinoa), where all samples were classified within the same statistical group (a). In contrast, mixed cereal matrices exhibited significant reductions in viability. In oat–buckwheat and buckwheat–quinoa samples, both 5% and 10% supplementation resulted in significantly lower viability compared to the control (a vs. b). In oat–quinoa samples, a significant decrease was observed only at 10% supplementation (a vs. b), indicating a dose-dependent effect.

IC_50_ values were calculated for all tested extracts. In most cases, the IC_50_ exceeded the maximum applied concentration (>2000 µg/mL), indicating very low cytotoxicity. Only three samples reached measurable IC_50_ values within the tested range: O2 (944.8 ± 6.4 µg/mL), OB2 (1447 ± 24 µg/mL), and OB3 (112.2 ± 1.8 µg/mL). These results demonstrate that the majority of extracts were non-toxic under the tested conditions, with moderate cytotoxic effects observed only for selected oat and oat–buckwheat variants.

## 3. Discussion

The present study evaluated the effect of enriching gluten-free (pseudo)cereal porridges with cell-free homogenate of the carotenogenic yeast *Rhodotorula toruloides*. The results demonstrate that yeast supplementation significantly modifies the nutritional and functional properties of (pseudo)cereal matrices by increasing the content of β-glucans, bioactive lipidic metabolites, and by influencing fatty-acid composition, antioxidant, and biological activity. The observed effects depended both on the cereal base and on the level of yeast supplementation, indicating that interactions between cereal components and microbial metabolites play an important role in determining the final functional profile of the porridges.

The relatively high standard deviations observed in some samples may be attributed to the heterogeneous nature of the cereal matrices and the presence of microbial biomass, which can affect extraction efficiency and analytical variability.

The β-glucan content observed in oat (7.78 g/100 g DW) corresponds to previously reported values for oats, typically ranging from 3–7% depending on cultivar and processing. Oat is therefore considered one of the main dietary sources of β-(1→3)(1→4)-glucans among cereals [39]. In contrast, pseudocereals such as buckwheat and quinoa contain only negligible amounts of β-glucans [40]. The relatively high β-glucan content detected in *R. toruloides* homogenate (16.45 g/100 g DW) reflects the structure of yeast cell walls composed mainly of β-(1→3)-glucans with β-(1→6) branching [29]. Yeast β-glucans are increasingly recognized as functional food ingredients due to their immunomodulatory and antioxidant properties [19]. A limitation of this study is that the β-glucan content in composite samples was not determined experimentally but estimated from the individual components, which may introduce uncertainty into the reported values and affect the robustness of comparisons between samples. Future studies should include direct quantification of β-glucans in composite samples.

The lipid-soluble metabolite profile of *R. toruloides* was consistent with previous reports, with some differences (mainly in lipid-soluble provitamins) likely resulting from cultivation conditions and/or homogenization and extraction procedures [41,42]. Cell disruption and heat inactivation increased carotenoid and sterol extractability, likely due to improved cell wall permeability [43]. Enrichment of porridges with *R. toruloides* homogenate significantly increased lipidic metabolites absent in controls. Ergosterol was the most abundant compound (>400 µg/g DW), highlighting the nutritional potential of *R. toruloides* as a precursor of vitamin D_2_ [32]. Carotenoids reached nearly 450 µg/g DW in samples with 10% RTh, with torularhodin as the dominant pigment [44]. The overall increase in metabolite concentrations with higher supplementation (10% vs. 5% RTh addition) indicates a dose-dependent effect and demonstrates the possibility of using *R. toruloides* cell homogenate as a natural source of sterols and carotenoids for functional food fortification. Such enrichment not only improves the nutritional profile of cereal-based products but may also enhance their antioxidant and potential antimicrobial activities, which are particularly relevant for gut health applications.

The cereal matrix influenced fatty-acid composition: quinoa provided high PUFA levels, whereas buckwheat favored MUFA [45,46]. The addition of *R. toruloides* shifted the profile toward MUFA and SFA (oleic and palmitic acids), improving oxidative stability due to reduced PUFA susceptibility to oxidation, although excessive SFA may reduce nutritional benefits [47,48].

Buckwheat porridges exhibited the highest antioxidant capacity, consistent with their high phenolic content (rutin, quercetin) [12]. Quinoa showed intermediate activity, while oats displayed lower values due to lower polyphenol content, despite the presence of avenanthramides [13,14]. The enrichment significantly increased antioxidant capacity, particularly at 5%, likely due to yeast-derived hydrophilic compounds such as β-glucans, mannans, peptides, and exopolysaccharides [30,49]. The slight decline at 10% may result from matrix interactions or limited solubility of these compounds [31,50]. These findings indicate that 5% RTh supplementation is optimal under the tested conditions for enhancing antioxidant capacity.

Approximate prebiotic potential depends on both cereal composition and yeast enrichment. Quinoa polysaccharides and phenolics stimulate bifidobacteria and lactobacilli, while oat β-glucans are well-known prebiotics improving cholesterol and glycaemia control [51,52]. It should be emphasized that the evaluation of prebiotic properties was based on a simplified model using two bacterial strains. Therefore, the results should be interpreted as indicative rather than definitive evidence of prebiotic functionality, and further studies involving more complex microbiota models are required.

The positive effect of 5% RTh is consistent with the prebiotic properties of yeast β-glucans and mannan-oligosaccharides [53,54]. The strongest stimulation was observed in the oat–quinoa combination, suggesting synergistic interactions between cereal and yeast polysaccharides. These findings underline the potential of quinoa and oat-based porridges, combined with moderate RTh enrichment, as promising carriers for prebiotic functional foods.

Cytotoxicity results indicated low toxicity overall. Samples without RTh showed minimal effects, whereas 5–10% supplementation moderately increased cytotoxic responses in some formulations. The moderate decrease in cell viability observed at the 10% supplementation level, particularly in buckwheat and oat–buckwheat samples, suggests that higher enrichment levels may negatively affect cytocompatibility, potentially due to increased concentrations of bioactive or matrix-associated compounds. In contrast, the 5% supplementation level generally maintained good cell viability while still providing functional benefits, suggesting that it may represent a more suitable balance between bioactivity and cytocompatibility. However, the buckwheat–quinoa combination maintained high cell viability regardless of RTh content, suggesting potential protective interactions between cereal components. These findings confirm that the biological effects of the porridges depend on both cereal composition and yeast supplementation level. The cytotoxicity evaluation in this study was performed using a single intestinal cell model (Caco-2), which represents a simplified system and does not fully capture the complexity of the gastrointestinal environment. Therefore, the obtained results should be interpreted with caution and should not be generalized as definitive evidence of safety. Further studies are required to confirm these findings using more advanced models, including additional cell lines, co-culture systems, in vitro digestion models, and sensory evaluation, in order to better assess the safety and practical applicability of the enriched products.

The observed differences in the response of individual cereal matrices to *Rhodotorula toruloides* enrichment indicate that the resulting compositional and functional changes are strongly matrix-dependent and cannot be explained solely by the amount of added biomass. These differences are likely governed by interactions between yeast-derived bioactive compounds and the specific macromolecular composition of the cereal substrates.

Oat-based samples showed distinct behavior compared to pseudocereal matrices, likely due to their high content of mixed-linkage β-glucans, which increase viscosity and may reduce the extractability of bioactive compounds [55], whereas quinoa and buckwheat differ in macromolecular composition and phenolic profile, which can influence the release and availability of antioxidants [56,57]. The addition of *R. toruloides* biomass further introduced yeast β-(1→3)(1→6)-glucans and lipophilic compounds, whose interactions with the matrix may affect the accessibility and apparent concentration of antioxidants [58,59]. The non-linear effect observed between 5% and 10% enrichment suggests that higher fortification does not necessarily improve extractability, possibly due to saturation or structural constraints within the matrix [58,60].

Overall, the results suggest that the measured antioxidant activity and related parameters reflect not only the total content of bioactive compounds but also their release and extractability from the food matrix. This highlights the importance of considering matrix effects when interpreting the functional properties of enriched cereal products. Future studies should aim to directly investigate these interactions using advanced analytical approaches, such as microscopy, binding studies, or in vitro digestion models, to better elucidate the underlying mechanisms.

In addition to the compounds evaluated in this study, yeasts are also known to produce B-group vitamins, which may further contribute to the nutritional value of fortified cereal-based products. However, this aspect was not investigated in the present work and may be addressed in future studies.

## 4. Materials and Methods

### 4.1. Selection of Cereals and Yeast Strain

Samples of cereals were selected for their gluten-free nature, price, availability, and nutritional composition. For these reasons, gluten-free oat flakes (Provita, Brno, Czech Republic), quinoa seeds (Provita, Brno, Czech Republic) and buckwheat seeds (Šmajstrla, Brno, Czech Republic) were chosen as a base of the cereal porridges.

The basic nutritional composition (proteins, lipids, carbohydrates, and simple sugars) of the cereal and pseudocereal raw materials used in this study was obtained from manufacturer-declared values (Table 5). These data were used to provide a general nutritional characterization of the raw materials forming the basis of the developed products. It should be noted that these values represent approximate composition and may vary depending on cultivar, processing conditions, and analytical methodology.

To enrich cereal porridges with bioactive substances such as antioxidants, essential fatty acids, carotenoids, and provitamin D, the yeast *R. toruloides* (RT) CCY 062-002-001 was chosen. The yeast strain was obtained from the Culture Collection of Yeasts at the Institute of Chemistry, Slovak Academy of Sciences, Bratislava, Slovakia.

### 4.2. Cultivation of R. toruloides

The yeast *R. toruloides* was initially cultivated on solid YPD agar medium, composed of 20.0 g/L glycerol, 10.0 g/L yeast extract, 20.0 g/L peptone, and 20.0 g/L agar (Roth, Germany) in distilled water. For liquid pre-cultures, the same medium formulation was used, omitting the agar. Five loops of the grown culture from Petri dishes were transferred into 50 mL of liquid YPD medium and incubated in Erlenmeyer flasks at room temperature on a reciprocal shaker at 100 rpm for 24 h. This pre-culture was subsequently transferred to fresh YPD medium in a 1:5 ratio and further cultivated under identical conditions for an additional 24 h.

The resulting inoculum was then introduced into a production mineral medium (46.46 g/L glycerol, 4.00 g/L KH_2_PO_4_, 0.696 g/L MgSO_4_, and 4.00 g/L (NH_4_)_2_SO_4_ (Lach-Ner, Neratovice, Czech Republic), all dissolved in 1000 mL of distilled water) at a 1:10 ratio and incubated for 96 h under aerobic conditions at room temperature and 100 rpm. The composition of the production mineral medium was selected based on previously published studies [61]. After cultivation, the yeast biomass was harvested by centrifugation. The cell suspension was frozen and stored at −20 °C. Before use, a water extract (suspension) was prepared and used for testing, as described below (paragraph 4.3.1).

### 4.3. Model Cereal Porridges

#### 4.3.1. Preparation of Model Porridges

Before preparing the model cereal porridges, the selected cereals were milled into flour using a grinder (SENCOR SCG 1050BK, SENCOR, Říčany, Czech Republic). For enrichment, RT biomass was applied in the form of cell-free homogenate (RTh). Cell disruption was performed by high-pressure homogenization, which is reproducible and scalable, generates uniform small particle sizes, and works with a wide range of viscosities. The advantages are chemical-free cell disruption and operation in a closed system, which results in a low risk of contamination [62]. After homogenization, the product was lyophilized and stored in darkness at −20 °C. Before use, a water extract (suspension) was prepared and used for testing.

The composition of the model porridges is shown in Table 6. Pure cereal porridges were labeled as type 1, and enrichment with RTh was applied at levels of 5% (type 2) and 10% (type 3). The dry mixtures were poured over with hot distilled water (90 °C), mixed thoroughly, and allowed to cool. This procedure was intended to simulate home preparation of instant porridges. The porridges were then lyophilized.

#### 4.3.2. Extracts from Model Cereal Porridges

For the analysis of model porridges and RTh in terms of antioxidant capacity and cytotoxicity, aqueous extracts were prepared. The samples were weighed and mixed with distilled water to achieve a final concentration of 100 mg/mL. To enhance extraction efficiency, glass beads with a diameter of 0.25–0.5 mm, Roth (Dautphetal, Germany) were added. The extraction was carried out for 1 h under vigorous shaking using a vortex mixer. The solid fraction was then separated by centrifugation, and the resulting aqueous extract was subjected to the above-mentioned analyses.

### 4.4. Composition of Extracts

#### 4.4.1. β-Glucans

The determination of β-glucans was performed using β-glucan assay kits (Megazyme, Bray, Ireland).

Extraction of β-glucans from cereals: The cereal samples weighing 80–120 mg were placed into test tubes, followed by the addition of 0.2 mL of 50% ethanol and 4 mL of phosphate buffer (20 mM, pH 6.5). The mixtures were vortexed and incubated in a water bath (100 °C, 60 s). Subsequently, the mixtures were vortexed again, incubated (100 °C, 2 min), and vortexed once more. Next, the mixtures were incubated in a water bath (50 °C, 5 min). Then, 0.1 mL of the enzyme lichenase was added, the mixtures were vortexed, and incubated (50 °C, 1 h). During incubation, the mixtures were vortexed several times. After incubation, 5 mL of acetate buffer (200 mM, pH 4.0) was added, the mixtures were vortexed and centrifuged (10,000 rpm, 10 min). To 0.1 mL of the obtained supernatants, 0.1 mL of β-glucosidase was added. The mixtures were then incubated (50 °C, 10 min).

Extraction of β-glucans from yeasts: The content of β-glucans in yeast was determined by measuring the content of α-glucans and total glucans. For the determination of α-glucans, 100 mg of yeast biomass was weighed into a test tube, and 2 mL of 1.7 M sodium hydroxide was added. The mixture was vortexed and placed in an ice bath for 20 min with occasional mixing. Subsequently, 8 mL of acetate buffer (1.2 M, pH 3.8) and 0.2 mL of an enzyme mixture (amyloglucosidase and invertase) were added. The mixture was vortexed and incubated (40 °C, 30 min). Then, 2 mL of the mixture was transferred into clean test tubes and centrifuged (13,000 rpm, 5 min). To 0.1 mL of the supernatant, 0.1 mL of acetate buffer (200 mM, pH 4.5) was added.

Analysis: For determination of total glucans, 90 mg of yeast biomass was weighed into a test tube and 2 mL of chilled 12 M sulfuric acid was added. The tube was placed in an ice bath for 2 h with occasional mixing. Subsequently, 4 mL of distilled water was added, and the mixture was vortexed (10 s), followed by the addition of another 6 mL of distilled water. The mixture was vortexed again (10 s). The tube, with the cap loosely closed, was placed in a water bath (100 °C, 2 h). After the first 5 min of incubation, the caps were fully closed. After cooling to room temperature, the contents of the tube were quantitatively transferred into a 100 mL volumetric flask, 6 mL of 8 M sodium hydroxide was added, and the flask was filled up to the mark with acetate buffer (200 mM, pH 4.5). The contents of the flask were mixed, and a 1 mL aliquot was transferred into a clean test tube and centrifuged (13,000 rpm, 5 min). To 0.1 mL of the supernatant, 0.1 mL of an enzyme mixture (exo-1,3-β-glucanase and β-glucosidase) in acetate buffer (200 mM, pH 4.5) was added. The mixture was vortexed and incubated (40 °C, 1 h). To the prepared extracts, 3 mL of GOPOD reagent was added and the mixtures were incubated (50 °C, 20 min). After incubation, the absorbance was measured at 510 nm against a blank. The β-glucan content in yeast biomass was calculated by subtracting the α-glucan content from the total glucan content.

#### 4.4.2. Lipid-Soluble Vitamins and Provitamins

Extraction: Lyophilized samples (model porridges, RTh, and RT) were rehydrated prior to extraction. The hydrated samples were then placed into microcentrifuge tubes containing glass beads (diameter of 0.25–0.5 mm, Roth, Dautphetal, Germany), chloroform and methanol (Penta, Praha, Czech Republic), and subjected to mechanical disruption using a laboratory vortex. This procedure facilitated cell lysis and enabled the extraction of lipid-soluble compounds, including carotenoids (provitamin A), ergosterol (provitamin D_2_ precursor) and other antioxidant molecules, following a modified Folch extraction protocol [42,63]. The resulting organic phase, with chloroform as the primary extraction solvent, was evaporated to dryness under a stream of nitrogen. The dry extracts were subsequently reconstituted in a 2:1 (*v*/*v*) mixture of HPLC-grade ethyl acetate and acetonitrile (Penta, Praha Czech Republic), in a volume suitable for high-performance liquid chromatography (HPLC) analysis with photodiode array (PDA) detection [42].

HPLC analysis: Quantitative and qualitative analysis of lipid-soluble compounds was performed using a high-performance liquid chromatography system (HPLC, Thermo Fisher Scientific, Waltham, MA, USA) equipped with a photodiode array (PDA) detector. Separation was carried out on an EVO C18 Kinetex column (150 mm × 4.6 mm, 2.6 μm particle size; Phenomenex, Torrance, CA, USA) under gradient elution. The mobile phase flow rate was maintained at 1.2 mL/min, and the total run time was 25 min. Mobile phase A consisted of methanol/acetonitrile: 100 mM Tris-HCl buffer (2:84:14, *v*/*v*), while mobile phase B was composed of methanol/ethyl acetate (60:40, *v*/*v*). The following gradient program was applied: 0–13 min, a linear transition from 100% A to 100% B; 13–19 min, held at 100% B; 19–20 min, return to 100% A; and 20–25 min, equilibration at 100% A. Detection wavelengths were set at 450 nm for carotenoids and 280 nm for sterols [42].

#### 4.4.3. Lipids and Fatty Acids

Extraction: Lyophilized samples (RTh, RT, and model porridges) were weighed into crimp-top vials, followed by the addition of 1.8 mL of a transesterification reagent consisting of 15% (*v*/*v*) sulfuric acid (Lach-Ner, Neratovice, Czech Republic) in methanol (Penta, Praha, Czech Republic), containing heptadecanoic acid (C17:0) as an internal standard at a concentration of 0.5 mg/mL. The vials were sealed and incubated in a thermoblock at 85 °C for 120 min to complete transesterification. After cooling to room temperature, the reaction mixtures were quantitatively transferred to 4 mL screw-cap glass vials. To facilitate phase separation and neutralization, 1 mL of HPLC-grade hexane (Penta, Praha, Czech Republic) and 0.5 mL of 0.05 M sodium hydroxide (Lach-Ner, Neratovice, Czech Republic) were added. The samples were vortexed for 10 min. The upper hexane layer—containing fatty acid methyl esters (FAMEs)—was collected in a new glass vial. The final volume was adjusted with hexane in proportion to the initial sample weight, and the extracts were analyzed by gas chromatography with flame ionization detection (GC-FID).

GC analysis: Fatty acid methyl esters were analyzed using a TRACE 1300 gas chromatograph (Thermo Scientific, Waltham, MA, USA) equipped with a flame ionization detector (FID), an AI 1310 autosampler, and an automatic split/splitless injector. Separation was achieved on a LION GC-FAME capillary column (30 m × 0.25 mm × 0.25 µm film thickness). The injector temperature was set to 240 °C with a split ratio of 1:10. The injection volume was 1 µL, and the carrier gas (helium) flow rate was maintained at 1.0 mL/min. The FID operated at 240 °C with the following gas flow rates: air at 350 mL/min, hydrogen at 35 mL/min, and nitrogen (make-up gas) at 30 mL/min. The oven temperature program was as follows: initial temperature 80 °C held for 1 min; ramped to 140 °C at 15 °C/min (t = 5 min); then to 190 °C at 3 °C/min (t = 21.7 min); and finally to 260 °C at 25 °C/min, held for 1 min, with a total run time of 25.5 min [42].

### 4.5. Characteristics of Cereals and Yeast Extracts

#### 4.5.1. Antioxidant Properties

To assess antioxidant properties, the ABTS·+ assay with Trolox standard was used. The ABTS radical was generated by reacting 7 mM ABTS (2,2′–azino–bis(3–ethylbenzothiazoline–6–sulfuric acid) diammonium salt) (Sigma-Aldrich, St. Louis, MO, USA) with 2.45 mM potassium persulfate (Sigma-Aldrich, St. Louis, MO, USA) and incubating the mixture in the dark for 12–16 h. Before analysis, the solution was diluted with UV-VIS ethanol (Penta, Praha Czech Republic) to an absorbance of 0.70 ± 0.02 at 734 nm. For the assay, 1 mL of diluted ABTS·+ was mixed with 10 μL of aqueous extract of model porridges and RTh, incubated in the dark, and absorbance was measured after 10 min. Results were expressed as Trolox equivalents using a standard curve (50–400 μg/mL) [64].

#### 4.5.2. Approximate Prebiotic Potential

To assess the approximate prebiotic potential, two lactic acid bacteria (LAB) strains, *Lactobacillus plantarum* CCM 7039 and *Bifidobacterium bifidum* CCM 3762, were selected as model probiotic microorganisms and obtained from the Czech Collection of Microorganisms (Brno, Czech Republic).

*B. bifidum*, originally isolated from human stool, is a well-established member of the human gastrointestinal microbiota, while *L. plantarum*, although originally isolated from fermented plant material, is commonly detected in the human gastrointestinal tract and is widely used as a model organism in studies evaluating prebiotic effects. The probiotic strains were cultivated in MRS broth (Carl Roth GmbH, Karlsruhe, Germany).

Cultivation of LAB: Both probiotic strains were cultivated for 24 h in MRS broth under anaerobic conditions at 37 °C. After incubation, cultures were adjusted to 1.5 × 10^8^ CFU/mL. A 10% (*v*/*v*) inoculum was transferred into 12 mL of a mixture consisting of model porridge (5% *v*/*v*) in MRS broth.

Evaluation of cell count: After cultivation, bacterial suspensions were centrifuged at 4500 rpm for 2 min. The resulting supernatant was diluted 1:10,000 with sterile phosphate-buffered saline (PBS, VWR, Stříbrná Skalice, Czech Republic). Subsequently, 100 µL of diluted culture was inoculated onto appropriate selective media (MRS agar) and incubated at 37 °C for 18 h. The number of colonies was subsequently counted using an automated colony counter (Scan 300, Interscience, Saint-Nom-la-Bretèche, France) with sensitivity set to 50% and diameter set to 0 mm. The prebiotic index (PI) was calculated as the ratio of bacterial growth in the test samples relative to that in MRS. The standard deviation was determined using standard error propagation rules for division. Although some samples showed high variability in prebiotic index, this is expected due to biological fluctuations typical for live microbial assays.

### 4.6. Cytotoxicity Assay by MTT Test

For cytotoxicity testing of the model porridges, the human colorectal adenocarcinoma cell line (Caco-2) was used. The Caco-2 cell line was obtained from the Cytion GmbH (Heidelberg, Germany). Caco-2 cells were cultivated in a sterile incubator at 37 °C, 5% CO_2_, and 95% relative humidity. The culture medium consisted of MEM (Sigma-Aldrich, St. Louis, MO, USA) supplemented with 10% fetal bovine serum (FBS, HyClone, Logan, UT, USA), 1% non-essential amino acids (NEAAs, Sigma-Aldrich, St. Louis, MO, USA), and 1% antibiotic solution (BioTech, Praha, Czech Republic). Cell cultures were supplied with fresh medium every 2–3 days and subsequently passaged when reaching approximately 80% confluence. All cell handling procedures were performed under strict sterile conditions [65].

Cytotoxicity of aqueous extracts from model porridges and RTh was evaluated using the MTT assay on the human Caco-2 cell line. Prior to testing, the extracts were prepared according to the established protocol [66].

First, 100 μL of properly diluted cell suspension was added to each well of a 96-well plate and incubated for 24 h. The medium was then replaced with the prepared sample dilutions in MEM to achieve a concentration range of 0.06–2 mg/mL and plates were returned to the incubator. MEM served as a negative control, while 25% DMSO (Roth, Dautphetal, Germany) was used as a positive control and 20% distilled water was used as a vehicle control. After an additional 24 h, the medium with samples was replaced with 20 μL of MTT solution (Sigma-Aldrich, St. Louis, MO, USA) (2.5 mg/mL in PBS) and plates were incubated for 3 h. Then, 100 μL of 10% sodium dodecyl sulfate (SDS, Serva, Heidelberg. Germany) in PBS was added to solubilize the formazan crystals. Plates were stored in the dark overnight and absorbance was measured the next day at 540 nm using an ELISA reader Synergy HTX, BioTek (Winooski, VT, USA) [64,66]. The results were evaluated in accordance with ISO 10993-5 [67].

### 4.7. Statistical Analysis

All measurements were performed in triplicate, and results are presented as mean ± standard deviation. Statistical differences among groups were evaluated by one-way analysis of variance (ANOVA), followed by Tukey’s post hoc test for multiple comparisons. Differences were considered statistically significant at *p* < 0.05. Statistical analysis was performed in Python 3.

## 5. Conclusions

This study demonstrates that enrichment of gluten-free cereal porridges with *Rhodotorula toruloides* cell homogenate significantly enhances their nutritional and functional properties. Supplementation, particularly at 5% RTh, increased antioxidant capacity, supported probiotic growth, and introduced bioactive lipidic compounds such as carotenoids and ergosterol, which are absent in unfortified porridges. Quinoa and buckwheat-based porridges exhibited superior antioxidant and prebiotic effects compared to oats, while the buckwheat–quinoa combination maintained high cell viability and showed minimal cytotoxicity. Although higher supplementation (10% RTh) further increased bioactive metabolites, it was associated with reduced probiotic stimulation and slightly higher cytotoxicity, suggesting that moderate cell homogenate addition provides the most balanced outcome. Overall, these results highlight *R. toruloides* cell homogenate as a promising fortifying ingredient for the development of gluten-free functional foods with improved health-promoting potential.

The combined features—antioxidant carotenoids, provitamin D_2_ potential, and health-promoting lipids—make *R. toruloides* one of the most attractive candidates for the fortification of cereal-based gluten-free food with nutritionally valuable bioactive compounds. Developing an efficient yeast production procedure and optimization of subsequent phases related to extraction, purification, and the preservation of their bioactive properties are crucial for the still underdeveloped use of yeasts in various industrial sectors.

## Figures and Tables

**Table 1:** Content of β-glucans; the results were expressed as g of β-glucans per 100 g of dry weight of model porridges.

Sample	Content of β-Glucans [g/100 g of DW]
oat	7.78 ± 0.41
oat + 5% RTh	8.60 ± 0.43
oat + 10% RTh	9.42 ± 0.47
buckwheat	0.17 ± 0.01
buckwheat + 5% RTh	1.00 ± 0.05
buckwheat + 10% RTh	1.82 ± 0.09
quinoa	0.27 ± 0.04
quinoa + 5% RTh	1.09 ± 0.05
quinoa + 10% RTh	1.91 ± 0.10
oat–buckwheat	3.98 ± 0.20
oat–buckwheat + 5% RTh	4.80 ± 0.24
oat–buckwheat + 10% RTh	5.62 ± 0.28
oat–quinoa	4.02 ± 0.20
oat–quinoa + 5% RTh	4.84 ± 0.24
oat–quinoa + 10% RTh	5.67 ± 0.28
buckwheat–quinoa	0.22 ± 0.01
buckwheat–quinoa + 5% RTh	1.04 ± 0.05
buckwheat–quinoa + 10% RTh	1.86 ± 0.09
*R. toruloides* homogenate	16.45 ± 0.10

**Table 2:** Content of lipid-soluble compounds; the results were expressed as µg of lipid-soluble metabolite per g of dry weight of model porridges; ND—not detected.

Amount of Lipid-Soluble Compounds [µg/g DW]					
	Ergosterol [µg/g]	Total Carotenoids [µg/g]	Torularhodin [µg/g]	Lycopene [µg/g]	Torulene [µg/g]
Oat	ND	ND	ND	ND	ND
Oat + 5% RTh	165.35 ± 6.88	ND	ND	ND	ND
Oat + 10% RTh	414.06 ± 42.09	298.62 ± 93.38	111.54 ± 9.59	69.41 ± 5.97	117.62 ± 0.24
Buckwheat	ND	ND	ND	ND	ND
Buckwheat + 5% RTh	227.77 ± 13.30	255.11 ± 7.27	70.08 ± 8.35	68.49 ± 5.21	116.54 ± 6.29
Buckwheat + 10% RTh3	355.49 ± 26.54	256.63 ± 29.97	71.60 ± 5.44	69.17 ± 5.60	115.86 ± 8.60
Quinoa	ND	ND	ND	ND	ND
Quinoa + 5% RTh	233.46 ± 9.28	213.99 ± 8.99	50.79 ± 4.37	47.37 ± 2.63	115.83 ± 10.64
Quinoa + 10% RTh	433.71 ± 15.85	390.02 ± 6.94	178.55 ± 3.26	102.79 ± 1.53	108.67 ± 8.67
Oat–buckwheat	ND	ND	ND	ND	ND
Oat–buckwheat + 5% RTh	201.69 ± 10.89	235.74 ± 12.16	70.73 ± 2.37	59.51 ± 2.07	105.50 ± 12.45
Oat–buckwheat + 10% RTh	392.78 ± 25.01	443.86 ± 0.85	214.24 ± 13.15	118.19 ± 0.95	110.98 ± 13.70
Oat–quinoa	ND	ND	ND	ND	ND
Oat–quinoa + 5% RTh	200.44 ± 15.91	211.78 ± 28.10	49.92 ± 3.29	52.31 ± 8.85	109.55 ± 4.70
Oat–quinoa + 10% RTh	394.17 ± 24.62	419.54 ± 55.34	155.91 ± 8.75	142.49 ± 13.11	121.14 ± 0.84
Buckwheat–quinoa	ND	ND	ND	ND	ND
Buckwheat–quinoa + 5% RTh	212.73 ± 9.88	225.86 ± 6.82	75.45 ± 9.81	52.85 ± 2.27	97.56 ± 5.26
Buckwheat–quinoa + 10% RTh	402.36 ± 4.73	429.85 ± 13.11	197.04 ± 4.26	121.11 ± 0.54	111.58 ± 9.53

**Table 3:** Content of fatty acids; the results are expressed as µg/mL.

Amount of Fatty Acids [µg/mL]						
**Fatty Acid**	**Oat**	**Oat + 5% RTh**	**Oat + 10% RTh**	**Buckwheat**	**Buckwheat + 5% RTh**	**Buckwheat + 10% RTh**
palmitic	187.07 ± 9.43	232.77 ± 12.56	209.25 ± 2.40	58.06 ± 2.48	56.34 ± 0.71	71.69 ± 3.93
stearic	ND	13.84 ± 0.69	24.15 ± 0.03	ND	7.31 ± 0.37	19.86 ± 0.99
oleic	356.23 ± 18.48	454.67 ± 23.52	396.92 ± 0,03	152.95 ± 7.78	146.24 ± 0.35	167.79 ± 8.33
linoleic	412.80 ± 21.73	481.78 ± 24.32	394.80 ± 3.42	201.07 ± 10.77	140.47 ± 4.25	142.15 ± 7.05
α-linolenic	ND	9.87 ± 0.49	7.73 ± 0.67	2.85 ± 0.14	2.27 ± 0.11	5.40 ± 0.27
**Fatty Acid**	**Quinoa**	**Quinoa + 5% RTh**	**Quinoa + 10% RTh**	**Oat–Buckwheat**	**Oat–Buckwheat + 5% RTh**	**Oat–Buckwheat + 10% RTh**
palmitic	81.19 ± 4.97	102.10 ± 5.09	100.80 ± 5.04	82.01 ± 6.33	146.91 ± 7.27	174.25 ± 8.73
stearic	ND	8.47 ± 0.42	18.29 ± 0.23	ND	12.05 ± 0.60	27.15 ± 1.68
oleic	179.42 ± 7.85	214.84 ± 11.79	234.48 ± 12.39	175.49 ± 6.12	297.41 ± 15.01	364.08 ± 18.74
linoleic	579.95 ± 26.38	541.56 ± 27.29	488.88 ± 24.13	219.07 ± 4.50	314.91 ± 16.02	343.93 ± 17.99
α-linolenic	40.01 ± 3.23	38.72 ± 1.93	35.43 ± 1.82	ND	6.48 ± 0.32	9.53 ± 0.48
**Fatty Acid**	**Oat–Quinoa**	**Oat–Quinoa + 5% RTh**	**Oat–Quinoa + 10% RTh**	**Buckwheat–Quinoa**	**Buckwheat–Quinoa + 5% RTh**	**Buckwheat–Quinoa + 10% RTh**
palmitic	118.52 ± 13.72	147.54 ± 14.27	170.31 ± 0.24	53.73 ± 2.75	95.87 ± 4.17	77.58 ± 0.29
stearic	ND	10.79 ± 0.67	25.98 ± 1.55	ND	13.06 ± 0.65	14.70 ± 0.27
oleic	235.51 ± 18.66	285.83 ± 25.08	363.30 ± 5.98	131.99 ± 14.76	219.36 ± 11.30	186.15 ± 2.13
linoleic	448.17 ± 27.88	469.09 ± 29.14	487.30 ± 20.65	330.72 ± 26.96	414.03 ± 21.37	293.80 ± 4.84
α-linolenic	18.81 ± 1.80	22.41 ± 1.19	24.65 ± 1.38	17.40 ± 0.87	25.43 ± 1.37	18.52 ± 0.72
**Fatty Acid**	**RT Homogenate**	**RT**				
palmitic	199.23 ± 8.37	265.72 ± 7.03				
stearic	121.73 ± 2.65	154.01 ± 13.17				
lignoceric	15.91 ± 0.41	19.89 ± 0.25				
oleic	455.29 ± 15.21	595.73 ± 7.18				
linoleic	173.29 ± 6.15	238.24 ± 14.71				
α-linolenic	26.39 ± 1.15	44.11 ± 2.07				

**Table 4:** Approximate prebiotic potential of model porridges expressed as prebiotic index (PI), defined as the ratio of bacterial growth in model porridges relative to growth in MRS medium. PI categories: − (0–1), + (1–1.5), ++ (1.5–2.5), +++ (≥2.5). x—no detectable bacterial growth.

	*Lactobacillus plantarum*	*Bifidobacterium bifidum*
oat	−	−
oat + 5% RTh	+++	+++
oat + 10% RTh	x	++
buckwheat	x	++
buckwheat + 5% RTh	+++	++
buckwheat + 10% RTh	++	−
quinoa	+++	+++
quinoa + 5% RTh	++	++
quinoa + 10% RTh	+	−
oat–buckwheat	++	+++
oat–buckwheat + 5% RTh	++	++
oat–buckwheat + 10% RTh	+++	−
oat–quinoa	+++	+++
oat–quinoa + 5% RTh	x	+
oat–quinoa + 10% RTh	+++	+
buckwheat–quinoa	++	+++
buckwheat–quinoa + 5% RTh	−	+++
buckwheat–quinoa + 10% RTh	+	+
*R. toruloides* homogenate	+	−

**Table 5:** Basic nutritional composition of cereal and pseudocereal raw materials used in this study (proteins, lipids, carbohydrates); values are based on manufacturer-declared nutritional information.

Sample	Carbohydrates [g/100 g]	Proteins [g/100 g]	Lipids [g/100 g]
gluten-free oat flakes	61.0	12.0	6.2
buckwheat seeds	73.8	7.4	1.8
quinoa seeds	60.0	16.0	7.0

**Table 6:** Scheme of the model porridges composition.

Samples	Type of Cereal	Amount of RTh Biomass [%*w*/*w*]
O1	GF-oat flakes	0
O2	GF-oat flakes	5
O3	GF-oat flakes	10
B1	Buckwheat grains	0
B2	Buckwheat grains	5
B3	Buckwheat grains	10
Q1	Quinoa grains	0
Q2	Quinoa grains	5
Q3	Quinoa grains	10
OB1	GF-oat flakes and buckwheat grains (1:1 ratio)	0
OB2	GF-oat flakes and buckwheat grains (1:1 ratio)	5
OB3	GF-oat flakes and buckwheat grains (1:1 ratio)	10
OQ1	GF-oat flakes and quinoa grains (1:1 ratio)	0
OQ2	GF-oat flakes and quinoa grains (1:1 ratio)	5
OQ3	GF-oat flakes and quinoa grains (1:1 ratio)	10
BQ1	Buckwheat grains and quinoa grains (1:1 ratio)	0
BQ2	Buckwheat grains and quinoa grains (1:1 ratio)	5
BQ3	Buckwheat grains and quinoa grains (1:1 ratio)	10

## Data Availability

The original contributions presented in this study are included in the article. Further inquiries can be directed at the corresponding author.

## Abbreviations

RT	*Rhodotorula toruloides*
RTh	*Rhodotorula toruloides* cell homogenate
DW	dry weight
MUFA	monounsaturated fatty acid
PUFA	polyunsaturated fatty acid
SFA	saturated fatty acid
EFSA	European Food Safety Authority
FDA	U.S. Food and Drug Administration
SCFA	short chain fatty acid
PI	prebiotic index
